# Major Surgery and Human Character : A Novel

**Published:** 1914-06-13

**Authors:** 


### THE PROFESSION OF MEDICINE.

### Major Surgery and Human Character: A Novel.

It would be very strange if contemporary fiction and drama had overlooked altogether the splendid material, both for plot and incident, which has been furnished in the varied and extraordinary developments of medical science during the last thirty years. There are, certainly, three or four good novels dealing with modern medicine and surgery, as well as several that are mediocre; and both Shaw and Barrie have constructed amusing comedies on medical follies, chaffing the profession in their respective veins of humour. But until Mr. Morley Roberts published his recent novel,* of which all Harley Street is talking, no first-rate modern novel had been founded on an essentially surgical theme. From the literary standpoint, this novel ranks high. The style is terse, but elegant; vivid, yet suggestive of much that is unsaid. Packed full of original thought, at the same time the book provokes readers into thinking for themselves.

It is, however, not the beauties of a really fine piece of prose writing that chiefly concern an institutional journal, but rather the plot and its development as dealing with the relations between surgeon and patient, and the "patient's point of view" of a nursing-home and of a major operation in it. Thomas Waring is a successful middle-aged professional man, who has made his way to eminence by years of work and ability. His worldly success and a somewhat tangled family life have made him hard and masterful in character. After suffering for some time a gradually increasing abdominal torment, Waring consults a physician, and is subsequently advised to see a surgeon; in short, an abdominal cancer is diagnosed, and an operation is decided upon.

The story opens with Waring's entrance into the operating theatre of a private nursing home, and with a graphic description of his impressions, or rather of his subsequent recollections thereof. A very fine description of the operation follows, in which the characters of several strongly contrasted types of medical men are drawn with skill and sympathy. The surgeon is, naturally enough, the principal figure of this group; but the physician, the anæsthetist, and the assistant are all carefully touched in as well. So life-like are these portraits that suspicion is sure to be aroused that they are drawn from life. But it is difficult to identify them, and it seems likely that they are either creations of the imagination or at any rate composites. When "The Doctor's Dilemma" set medical tongues wagging, each of several large medical schools immediately claimed Sir Bloomfield Bonnington as one of its own senior physicians: a tribute either to Mr. Shaw's knowledge of human nature or to the prevalence of pretentious humbugs among titled specialists. We doubt whether Renshaw, the surgeon who operated on Thomas Waring, will be instantaneously identified in the schools; but he is very human, tremendously keen on surgery, and represents in many ways a fairly common, as well as favourable, type of the modern surgeon.

After the description of the operation, an exceedingly radical dissection of growth and glands from deep in the abdomen, there follows a graphic account of Waring's recovery from anæsthesia, and of the terrible sensations he experienced while in a state of profound surgical shock. The nurses who tended him are carefully drawn, though there is less detail in the picture than in that of the doctors. Both the institution and the nurses in it are efficient and capable; there is nothing of the querulous in this patient's point of view. After a period of intense depression, mental and physical, Waring gradually recovers, though the process is slow. As he gets stronger he begins to realise that many of his opinions have changed, and, in fact, his history is that of an altered man. His old strength is gone, and with it much of his hardness. He feels that to make his family and his friends happy is the best thing life offers him; whereas formerly his work and his reputation have held the foremost place in his thoughts. The manner of Waring's adoption of the creed of happiness, and the methods by which he puts it into execution, are described with rare skill. To unfold the developments of the plot in any further detail would not be fair to Mr. Roberts; these we leave his readers to find out for themselves.

The relations of Waring to his professional advisers; the extraordinarily imaginative analysis of Waring's surgical shock; and the steps of his protracted convalescence are of very great professional interest. So eloquent, so intimate, so convincing is this account of the results of major surgery that every operator ought to digest it in the interests of his future patients. It is sometimes said that every surgeon ought to undergo at least one laparotomy, so as to realise the feelings of his patients afterwards, and to spur him on to never-ending efforts to alleviate them. That is, perhaps, a counsel of perfection; but an excellent, if less drastic, substitute is provided by the descriptions of the patient's recovery in this novel.

The other great lesson which members of the medical profession may derive from the story of Thomas Waring is the realisation of what malignant disease may mean to an intelligent man, well aware of what his future may be. Statistics of the operative treatment of malignant disease teem with phrases such as "recurrence after two years," each of which is a summary in two or three words of a most moving and pitiable tragedy. If there ever is a tendency among surgeons (or physicians) to think more of the "case," the technique of operation, or other detail of treatment, than of the grim shadow in which their patient may be walking, of his hopes and doubts and fears, Mr. Morley Roberts's book is a most wholesome corrective.

---

* *Time and Thomas Waring.* (Eveleigh Nash. 1914. Price 6s.)

